# Impact of a digital employer-based weight loss program on individuals age 65 or older

**DOI:** 10.3389/fragi.2024.1337418

**Published:** 2024-05-22

**Authors:** Renee J. Rogers, Michael Doherty, David Jones, John M. Jakicic, Timothy S. Church

**Affiliations:** ^1^ Department of Internal Medicine, Division of Physical Activity and Weight Management, University of Kansas Medical Center, Kansas City, KS, United States; ^2^ Wondr Health, Dallas, TX, United States

**Keywords:** weight loss, obesity, aging, intervention, digital health

## Abstract

**Introduction:**

Older adults are not protected from obesity, which has been linked to frailty, cognitive impairment, and other aging-related factors. Intensive lifestyle interventions have been shown to be effective for weight loss in older adults; however, these have typically been highly intensive and less feasible for dissemination. This analysis describes weight loss in a large-scale, commercially available, digital intervention in a subset of older adults.

**Methods:**

Older adults (N = 20,443, males = 6,238; females = 14,205) between 65 and 85 years of age with overweight (43.3%) or obesity (46.7%) participated in an online, self-directed weight loss program. Behavioral-based content was delivered through weekly video lessons within an online platform that included weight and physical activity tracking, an online community, a reference library, and access to coaching support. Self-reported measures taken at the time of entry into the program were used for this analysis (demographics, height, body weight, and health status). Weight was reported across weeks of engagement in the curriculum.

**Results:**

The average weight loss was −3.15 kg (95% CI: [−3.20, −3.11]) at 15.5 weeks. Weight loss was significantly greater in male individuals (−3.79 kg [95% CI: −3.89, −3.71]) *versus* female individuals (−2.87 kg [95% CI: −2.94, −2.82]) (*p* < 0.001), with a similar engagement in curriculum weeks. Percent weight loss was statistically significant for all age categories (*p* < 0.05) and self-reported health conditions (*p* < 0.05).

**Discussion:**

Short-term weight loss was observed in older adults exposed to a low-touch, self-guided, and digital behavioral-based weight loss program. Weight loss was also observed even in the presence of various chronic health conditions.

## 1 Introduction

Obesity continues to be a major public health concern due to its high prevalence and its association with chronic health conditions. These include, but are not limited to, cardiovascular disease, diabetes, some forms of cancer, musculoskeletal disorders, and mental health concerns (Jensen et al., 2014; Bray et al., 2018). Of significant concern, older adults are not protected from obesity and its deleterious health effects, with recent estimates of more than 40 percent of older adults classified as obese in the United States (Hales et al., 2020). Excess body weight in older adults has been linked to frailty, cognitive impairment, and other aging-related factors (Miller and Spencer, 2014; Crow et al., 2019; Villareal, 2023). This supports the need for interventions to promote sustainable weight loss in older adults. Moreover, it is important to understand the response to various interventions for weight loss in older adults as this may affect the types of programs offered specifically to individuals in this age group. This may have implications for access to care and financial coverage for intervention approaches targeting weight loss treatment.

Intensive lifestyle interventions have been shown to reduce body weight and improve various health outcomes in older adults. For example, the age-group analysis of the Diabetes Prevention Program showed that the intervention was equally effective for weight loss across all age groups and, compared to metformin for the prevention of type 2 diabetes, was more effective in older adults (≥60 years of age) than people of younger ages (Knowler et al., 2002). The intensive lifestyle intervention for Look AHEAD resulted in significantly greater weight loss at 1 year in older adults (65–74 years of age) compared to those of younger ages (Wadden et al., 2009). However, these interventions were conducted within the context of structured university-based research centers and involved intensive in-person individual or group-based sessions, which may not be feasible for broader dissemination.

Alternative delivery approaches to these interventions have been examined. For example, the MOVE-UP study was a community-based intervention based on Look AHEAD delivered by community healthcare workers. This was applied to older adults (∼67 years of age) and resulted in a median weight loss of 5% of baseline weight (Albert et al., 2022). This study also showed that 5% weight loss was associated with improvements in mobility and function. Alternative deliveries of the Diabetes Prevention Program that have included various digital and mobile approaches have also shown effectiveness for weight loss but have not been examined specifically in older adults (Fukuoka et al., 2015). Broadly available commercial weight loss interventions have reported the inclusion of older adults; however, these studies have not reported on the effectiveness of these programs for this population (Tate et al., 2022b). Thus, there is a need to examine dissemination of behavioral-based weight loss interventions that can be applied on a broad scale to older adults with obesity.

This analysis examined weight loss in a large-scale, commercially available, self-guided digital intervention in a subset of adults ≥65 years of age. Additionally, weight loss was examined based on the presence of selective cardiometabolic risk factors and other health conditions.

## 2 Materials and methods

### 2.1 Participants

This analysis describes the weight loss outcomes of a large sample of 20,443 older adults with overweight or obesity who participated in an online, commercial weight loss program for company employees from March 2018 to November 2021. Companies provided program participation information to their employees via emails, post cards, and flyers at worksites. This was required for all workers, regardless of age. If benefits were retained after retirement, they were contacted through the appropriate entity providing these benefits. Participants from all regions of the continental United States voluntarily engaged in the program. This analysis used a de-identified dataset, and therefore, the requirement to document informed consent was waived, and the study was determined to be exempted from IRB oversight, according to the tenets of the US Department of Health and Human Services regulations at 45 CFR 46.

### 2.2 Program curriculum

The weight loss program (Wondr Health™, formerly Naturally Slim, Dallas, TX, USA) is a completely digital intervention delivered on a web-based distance learning platform. This platform allowed participants to flexibly engage at their convenience from any location with Internet access. Content was delivered through video lessons released each week across a period of 52 weeks, with each week having 20–30 min of material. The program curriculum was designed with constructs found in standard behavioral weight loss interventions such as self-monitoring, goal setting, stimulus control, modification of eating habits, and problem solving with additional focus on evaluating hunger signals and mindful eating. The focus of the core curriculum included the following: 1) mindful eating and portion control, stimulus control, medical considerations, and weight Loss; 2) stop eating cues and introduction to physical activity; 3) stress and emotions, mindless eating, goal setting, and problem solving physical activity; 4) hidden sugar, mindful activities, and energy balance; 5) nutrition 101, stress management, physical activity, and weight maintenance; 6) weight fluctuations, food cravings vs. easily accessible food, and Centers for Disease Control and Prevention (CDC) exercise recommendations; 7) emotions and eating, importance of self-monitoring, and making exercise a habit; 8) grocery shopping and meal planning, metabolic syndrome, and cognitive behavioral techniques; 9) serving sizes, social support, dealing with saboteurs; and 10) review of eating skills and tools, maintaining motivation, and long-term action planning. An outline of all program objectives has been previously published (Earnest et al., 2019). The platform also includes a participant weight and physical activity tracking area, online support community, a reference library, and access to coaching to answer questions.

Recommendations for dietary modification did not focus on eliminating specific foods or macronutrients but did recommend reducing refined sugar and carbohydrate intake while maintaining protein consumption at 25%–30% of total calories. However, the focus of the dietary approach is based on the Mediterranean and DASH diets to promote a balanced nutrient intake. The program curriculum also encouraged participation in moderate-intensity physical activity, such as brisk walking, based on the NIH consensus development panel on physical activity and cardiovascular health (NIH Consensus Development Panel on Physical Activity and Cardiovascular Health, 1996). The physical activity components were developed to promote weight loss and cardiometabolic health, and therefore, the physical activity was not specifically tailored to include activity specific for older adults (e.g., balance training).

### 2.3 Outcome measures

Demographic characteristics are based on a self-report by the participant at the time of enrollment in the program. Demographic characteristics include age, sex, and the presence of selective self-reported health conditions (hypertension, diabetes status [type 2 diabetes mellitus and pre-type 2 diabetes mellitus], non-alcoholic fatty liver disease, low high-density lipoprotein (HDL), elevated triglycerides, osteoporosis, and sleep apnea). Height and weight were also reported at baseline, and these data were used to compute body mass index (BMI, kg/m^2^). Moreover, weight was reported across weeks of engagement in the curriculum, which was used to compute absolute and percentage weight changes.

### 2.4 Statistical analysis

Statistical analysis was conducted using SAS on demand for academics. Statistical significance was *a priori* defined as *p* ≤ 0.05. Demographic characteristics are presented as mean ± standard deviation for continuous variables and as percentages for categorical variables. Absolute weight change and percent weight change were compared between male and female individuals using general linear modeling (GLM), with gender as a class variable. The GLM procedure uses the method of least squares to fit general linear models. Among the statistical methods available in PROC GLM are regression, analysis of variance, analysis of covariance, multivariate analysis of variance, and partial correlation. These analyses were repeated and included only those participants who completed ≥9 curriculum sessions, which is modeled after the same cut point used in prior published analyses (Earnest and Church, 2020). Percentage weight change adjusted for gender is presented as mean [95% confidence interval] across the number of weeks in the program (1–4, 5–8, 9–12, 13–16, and 17+).

Percentage weight change is also presented by age categories (65–69, 70–74, 75–79, and 80–85 years) and by the presence of self-reported health conditions. These data are presented for the entire sample and also by participants who completed ≥9 curriculum sessions. It has also been shown that a program based on the Diabetes Prevention Program attending nine or more sessions during the weight loss program was associated with improved outcomes related to the participant’s willingness to take action related to their weight loss behaviors, and therefore, this cut-point was adopted for the analyses in this study (Skrine Jeffers et al., 2019). By week 9 of the current intervention, participants had viewed content and practiced foundational weight management skills around awareness of hunger and satisfaction signals, basic nutrition, and meal planning, along with strategies to increase physical activity, improve sleep quality, manage stress and food cravings, elicit social support, and address challenges around emotional eating, social situations, and dichotomous thinking related to weight loss efforts. These data are presented as mean 95% confidence interval, with confidence intervals for the weight change that do not contain 0 as a value being significant at *p* < 0.05.

## 3 Results

The demographic characteristics of the study sample are shown in Table 1. In general, the participants were 68.7 ± 3.8 years of age with a BMI of 32.0 ± 5.3 kg/m^2^ (43.3% overweight and 46.7% obese) and primarily female individuals (69.5%). The most prevalent chronic health condition was hypertension (64% of participants).

**TABLE 1:** Descriptive characteristics of the study sample.

	All participants	Female	Male
Sample (N)	20,443	14,205	6,238
Age (years)*	68.7 ± 3.8	68.6 ± 3.7	68.9 ± 4.0
Weight (kg)*	90.8 ± 17.61	85.6 ± 16.1	100.3 ± 17.0
BMI (kg/m^2^)*	32.0 ± 5.3	32.3 ± 5.6	31.4 ± 4.7
Hypertension**	64%	61%	69%
Diabetes status			
Type 2 diabetes mellitus**	16%	15%	19%
Pre-Type 2 diabetes mellitus**	21%	21%	21%
Non-alcoholic fatty liver disease**	5%	6%	4%
Low HDL**	37%	36%	41%
Elevated triglycerides**	37%	35%	40%
Osteoporosis**	48%	54%	37%
Sleep apnea**	28%	24%	39%

*Data presented as mean ± standard deviation.

**Data presented as the percent of participants within the category with this diagnosed condition.

For the entire sample, based on the difference between the last weight obtained and baseline weight, weight loss measured was −3.15 kg (95% CI: [-3.20, −3.11]), and this was achieved following an average of 15.5 weeks of participation in the curriculum (Table 2). Weight loss was significantly greater in male individuals −3.79 kg (95% CI: [-3.89, −3.71]) *versus* female individuals (−2.87 kg [95% CI: −2.94, −2.82]) (*p* < 0.001), with male individuals completing 15.6 and female individuals completing 15.5 weeks of the curriculum. Similar findings were observed when the data were examined for percent weight loss. These analyses were repeated for participants who completed ≥9 weeks of the curriculum, with this being 19.2 weeks for male individuals and 19.7 weeks for female individuals. The weight loss expressed as changes in kg or percent weight change remained significantly greater in male individuals compared to female individuals (see Table 2).

**TABLE 2:** Absolute and percent weight change for the entire sample, participants, and participants who completed ≥9 curriculum sessions.

*Complete Study Sample*				
Variable	All participants (N = 20,443)	Female participant (N = 14,205)	Male participant (N = 6,238)	*p*-value*
	Mean (95% CI)	Mean (95% CI)	Mean (95% CI)	
Weight change, kg**	−3.15 (−3.20, −3.11)	−2.87 (−2.94, −2.82)	−3.79 (−3.89, −3.71)	<0.001
% Weight change	−3.48 (−3.53, −3.42)	−3.35 (−3.29, −3.41)	−3.77 (−3.67, −3.86)	<0.001
Week achieved	15.5	15.5	15.6	

*PARTICIPANTS WHO COMPLETED ≥9 CURRICULUM SESSIONS*				
Variable	All Participants (N = 13,700)	Female participant (N = 9,291)	Male participant (N = 4,409)	*p*-value*
	Mean (95% CI)	Mean (95% CI)	Mean (95% CI)	
Weight change, kg**	−4.03 (−4.10, −3.97)	−3.7 (−3.78, −3.62)	−4.73 (−4.89, −4.62)	<0.001
% Weight change	−4.44 (−4.51, −4.38)	−4.32 (−4.41, −4.24)	−4.70 (−4.81, −4.57)	<0.001
Week achieved	19.6	19.7	19.2	

*p-value for the comparison between female and male participants.

**Weight change is defined as the last available weight recorded minus the baseline weight.

**FIGURE 1:**
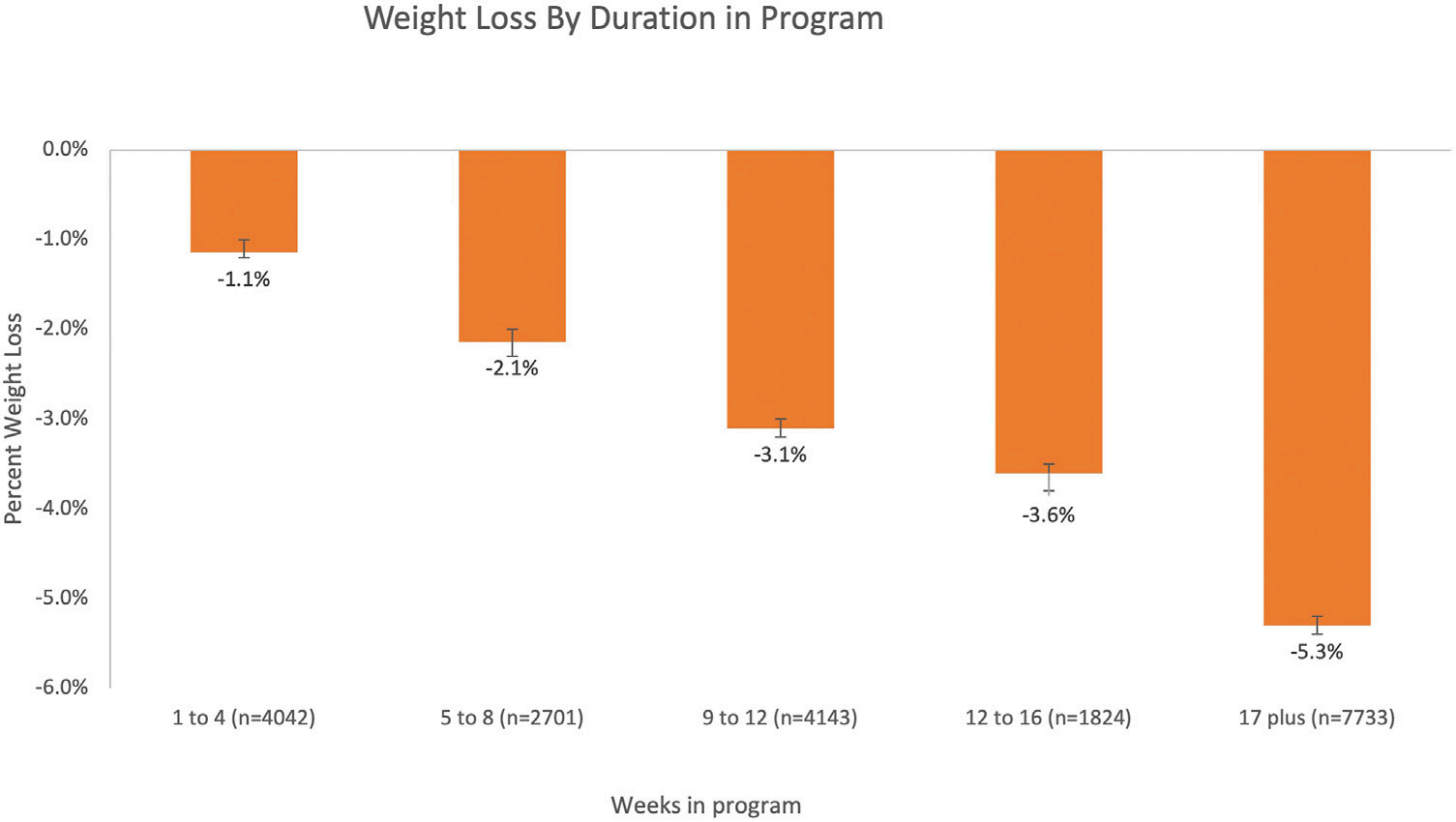
Percent weight loss (mean [95% CI]) by duration of participation in the program adjusting for age and gender.

The data were further examined by weeks of participation in the program adjusted for age and gender (Figure 1). Weight loss increased across program participation from 1.1% with engagement in 1–4 sessions to 5.3% with engagement in 17 or more sessions.

Percentage weight loss obtained from the final measured weight was examined by age category controlling for sex (Table 3). Percent weight loss was statistically significant (*p* < 0.05) for all age categories. A similar pattern of results was observed when only participants who completed ≥9 curriculum sessions were examined.

**TABLE 3:** Percentage weight change by age category and the presence of selective health conditions for all participants and for participants completing ≥9 curriculum sessions.

Participant characteristic		All participants		Participants completing ≥9 curriculum sessions	
		N	Mean (95% CI)^a^	N	Mean (95% CI)^a^
**Age category**	65–69 years	13,884	−3.43 (−3.49, −3.37)	9,330	−4.36 (−4.44, −4.28)
	70–74 years	4,681	−3.57 (−3.68, −3.46)	3,120	−4.61 (−4.76, −4.47)
	75–79 years	1,469	−3.67 (−3.87, −3.47)	982	−4.65 (−4.91, −4.39)
	80–85 years	409	−3.48 (−3.85, −3.10)	268	−4.66 (−5.16, −4.17)
**Self-reported health condition**	Hypertension	13,001	−3.43 (−3.50, −3.36)	8673	−4.40 (−4.48, −4.31)
	Diabetes status				
	• Pre-Type 2 diabetes mellitus	4,221	−3.15 (−3.27, −3.03)	2731	−4.10 (−4.26, −3.95)
	• Type 2 Diabetes mellitus	3,355	−2.99 (−3.12, −2.86)	2130	−3.92 (−4.09, −3.74)
	Elevated triglycerides	7,480	−3.44 (−3.53, −3.36)	4973	−4.42 (−4.54, −4.31)
	Low HDL	7,629	−3.37 (−3.46, −3.29)	5056	−4.36 (−4.47, −4.24)
	Sleep apnea	5,792	−3.23 (−3.34, −3.13)	3741	−4.23 (−4.37, −4.10)
	Osteoporosis	9,908	−3.42 (−3.50, −3.34)	6395	−4.44 (−4.55, −4.34)

^a^ Percentage weight change controlling for gender.

Percentage weight loss obtained from the final measured weight was also examined by different self-reported health conditions controlling for sex (Table 3). Percentage weight loss was statistically significant (*p* < 0.05) for all the health conditions examined. A similar pattern of results was observed when only participants who completed ≥9 curriculum sessions were examined.

## 4 Discussion

This study reports on the weight change observed, following a large-scale, commercially available, self-guided digital intervention applied in a subset of adults ≥65 years of age. Key findings from this study include that weight loss occurred in all age strata, in all chronic health conditions examined, was greater in male vs. female individuals, and was greater as engagement in the study was lengthened.

This study demonstrated the overall weight loss of −3.15 kg (95% CI: −3.20, −3.11), and this was observed at a mean exposure of 15.5 weeks of intervention. The weight loss improved to −4.03 kg at a mean of 19.6 weeks for participants who completed at least 9 sessions of the curriculum. By comparison, the Diabetes Prevention Program was delivered by DVD, which is similar to the video-based curriculum evaluated in this study, and 12-week weight loss of −5.4 kg was observed in adults with a mean age of 59.7 years (Kramer et al., 2010). When implemented in adult senior/community centers, the Diabetes Prevention Program demonstrated a 6-month weight loss of −5.4 kg in adults with a mean age of 62.8 years (Kramer et al., 2018). The 6-month weight loss in an in-person behavioral intervention delivered by community health workers to older adults (mean age = 67.7 years) was approximately 5% of baseline weight (Albert et al., 2022). Similar to this current study, these weight losses reflect the change from baseline in response to these interventions and not the difference compared to a comparison group.

In comparison to other commercial weight loss programs, a 12-week online weight loss intervention resulted in the weight loss of −2.7 kg compared to −1.3 kg in a newsletter control condition (Thomas et al., 2017). However, when delivered as an in-person intervention, weight loss was −4.9 kg at 6 months (Rogers RJ et al., 2020), and others have reported that this commercial program resulted in −3.8 kg at 3 months (Tate et al., 2022b), and these weight losses reflect the change from baseline. However, although these other studies recruited participants across a broad age range, weight loss results were not presented by age category, and therefore, the effectiveness of these other commercial approaches on weight loss in older adults is unclear. In the current study, weight loss was significant in the age categories of 65–69, 70–74, 75–79, and 80–85 years. It is also important to note that significant weight loss was also observed even in the presence of any of the chronic health conditions examined in this study.

The data presented represent weight loss in older adults. To provide a comparison, an in-person weight loss intervention with young adults (18.5–35.9 years) demonstrated a median weight loss of −7.8 kg at 6 months (25th percentile = −12.2 and 75th percentile = −3.7 kg) (Jakicic et al., 2015). In another study of young adults (29.4 ± 4.3 years), a 6-month personal coaching intervention resulted in a −1.92 kg (95th percent CI: −3.17 to −0.67 kg) weight change compared to control (Svetkey et al., 2015). In a study of middle-aged adults (50.9 ± 11.0 years), a 6-month Internet-based intervention resulted in a −4.49 kg (S.E. = 0.38 kg) weight change (Tate et al., 2022a). This may suggest that the weight loss observed in older adults in response to a self-guided digital intervention in the current study is blunted compared to what might be observed in younger age groups using different intervention platforms.

An important finding of this study was that weight loss improved as engagement in the intervention increased. These findings are consistent with other behavioral interventions for weight loss that demonstrated an association between a greater intervention engagement and greater weight loss (Wadden et al., 2009; Jakicic et al., 2015). Stevens et al. suggested that a weight change of <3% reflects weight stability, whereas reducing weight by at least 3% reflects weight loss, and this was achieved in this study when engagement in the intervention was at least 9–12 weeks in duration (Stevens et al., 2006). Moreover, a weight loss of at least 5% has been suggested to reflect a clinically meaningful weight loss, and this was achieved when the engagement in the intervention was at least 17 weeks. This may reflect the need to examine strategies for retaining engagement in this type of weight loss intervention for at least these durations to achieve a meaningful weight loss in older adults.

There are numerous strengths to this study that warrant recognition. This is a study of a commercially available, self-guided digital intervention and reports on weight loss observed in older adults. This study also included a large sample of older adults, both men and women, and participants with various health conditions, which may reflect real-world engagement in a weight loss intervention. However, despite these strengths, there are limitations that warrant consideration. The outcome data on weight along with the presence of chronic health conditions are based on the self-report. Moreover, weight rather than body composition data is available, and therefore, it is not possible to determine the contribution of lean body mass and adipose tissue to weight loss, which may be clinically important for older adults. This study also used an observational, non-randomized design and did not include a non-intervention comparison condition. Data are not available to determine the level of engagement of the participant while viewing the videos, and measures of dietary intake, eating behavior, and physical activity are not available to determine how these factors may contribute to the outcomes observed.

In summary, weight loss was observed in older adults 65–85 years of age with exposure to a commercial program that is low-touch, self-guided, and digital behavioral-based. This study also showed that a longer engagement in the program was associated with greater weight loss, suggesting the need for strategies to enhance the engagement of participants in this type of program. Of potential clinical importance, weight loss was also achieved even in the presence of various chronic health conditions. These findings warrant replication along with future studies to understand how the magnitude of intentional weight loss observed in this study may be associated with reducing chronic health conditions in older adults.

## Data Availability

The raw data supporting the conclusion of this article will be made available by the authors, without undue reservation.
